# A Slowly Varying Spoofing Algorithm on Loosely Coupled GNSS/IMU Avoiding Multiple Anti-Spoofing Techniques

**DOI:** 10.3390/s22124503

**Published:** 2022-06-14

**Authors:** Yangjun Gao, Guangyun Li

**Affiliations:** 1 College of Geospatial Information, PLA Strategic Support Force Information Engineering University, Zhengzhou 450001, China; yangjun_gao@163.com; 2 State Key Laboratory of Geo-Information Engineering, Xi’an 710054, China

**Keywords:** slowly varying spoofing, loosely coupled GNSS/IMU, anti-spoofing techniques, innovation sequence monitoring, rationality check

## Abstract

When satellite navigation terminal sensors encounter malicious signal spoofing or interference, if attention is not paid to improving their anti-spoofing ability, the performance of the sensors will be seriously affected. The global navigation satellite system (GNSS) spoofing has gradually become a research hotspot of the jammer because of its great harm and high concealment. In the face of more and more sensors coupling GNSS and inertial measurement unit (IMU) to varying degrees and configuring a variety of anti-spoofing techniques to effectively detect spoofing, even if the spoofer intends to gradually pull the positioning results, if the spoofing strategy is unreasonable, the parameters of the coupled filter output and spoofing observation measurement will lose their rationality, which will lead to the spoofing being detected. To solve the above problems, in order to effectively counter the non-cooperative target sensors of assembling loosely coupled GNSS/IMU using GNSS spoofing, based on the analysis of the influence mechanism of spoofing on the positioning of loosely coupled GNSS/IMU, a slowly varying spoofing algorithm to avoid loosely coupled GNSS/IMU with multiple anti-spoofing techniques is proposed in this paper, and a measurement deviation determination method to avoid multiple anti-spoofing techniques is proposed, which can gradually pull the positioning results of the coupled system and successfully avoid the detection of anti-spoofing techniques of innovation sequence monitoring and a rationality check on parameters. Simulation experimental results show that the proposed algorithm gradually changes the positioning of loosely coupled GNSS/IMU, the north and east displacements achieve the purpose of spoofing, and error with expected offset is −0.2 m and 2.3 m, respectively. Down displacement also basically achieves the purpose of spoofing, and error with the expected offset is 13.2 m. At the same time, the spoofer avoids the detection of multiple anti-spoofing techniques, does not trigger the system alarm, and realizes the purpose of spoofing; thus, the effectiveness and high concealment of the spoofing algorithm are verified.

## 1. Introduction

The global navigation satellite system (GNSS) spoofing technology has gradually become the focus of interference technology research because of its great threat and high concealment [1,2]. Spirent can manufacture a simple spoofing source by configuring the corresponding power amplifier and transmitting antenna on its product, GSS8000 [3]. In 2002, Jon S. Warner et al. used a simple GPS signal simulator to spoof a GPS receiver of a freight truck, demonstrating that the civil GPS receiver is vulnerable to a simple spoofing attack [4]. In 2012, Todd E. Humphrey’s team used a low-cost GPS spoofer to continuously lower the unmanned helicopter that should have maintained a fixed altitude [5]. In addition, GNSS spoofing can seriously affect GNSS timing information [6,7,8].

At present, there are many spoofing detection methods based on the single GNSS module [9,10], but any method is difficult when dealing with all spoofing methods [11]. In GNSS and the inertial measurement unit (IMU) system, IMU constantly uses measurement information of GNSS to correct its own error. Slow spoofing can also spoof the GNSS/IMU system, for example, traction spoofing can take over the loop without destroying the tracking loop [12]. When the difference between the position velocity and time (PVT) and real PVT is large, the user can detect spoofing by comparing with the measurement results of other sensors. Therefore, it is necessary to gradually pull PVT results to make the variation within the allowable range of the sensor error [13].

The research on the spoofing coupled system is summarized as follows. Mi Shi’s theoretical derivation and simulation show the influence of GNSS spoofing on the positioning results of loose coupling, but the mathematical model is not consistent with the actual situation that the target will adjust the trajectory after being affected by spoofing [14]. Yang Liu et al. studied the influence of GNSS spoofing on the Kalman filter error covariance matrix, innovation sequence and inertial sensor deviation estimation of a loosely coupled GNSS/INS system; their simulation showed that the Kalman filtering error covariance is not affected by spoofing, and innovation sequence and estimated inertial sensor deviation change [15]. Rui Xu et al. analyzed the performance of the position fusion and position/velocity fusion loosely coupled GNSS/IMU system under forwarding spoofing and traction spoofing. Under forwarding spoofing, the error compensation of the position fusion loosely coupled system for IMU is very significant, resulting in a jump in the positioning results. Under intermediate spoofing, the compensation increment of the loosely coupled system based on position/velocity fusion is more sensitive [16]. The navigation system adopts normalized innovation squared (NIS) detection; it is a direct, effective and feasible spoofing detection method, which is mature and has been applied to the navigation system of unmanned aerial vehicles (UAVs) and other targets [17]. Gao Yangjun et al. analyzed the influence of spoofing on the positioning of loosely coupled GNSS/IMU. Aiming at the problem that the equation is easy to be ill conditioned when the measurement deviation is introduced, a Kalman gain matrix local regularization method is proposed to accurately calculate the measurement deviation; in order to avoid the NIS detection alarm of the target navigation system, the range of measurement deviation is calculated, so that the spoofing process has strong concealment; then a two-step trajectory guidance algorithm is proposed so that the target can be quickly induced to the spoofing trajectory [18]. The above comprehensive literature review shows the representative research work on spoofing loosely coupled GNSS/IMU systems. After the summary, the difficulties of the current spoofing on a coupled system are described.

The background and motivation of this research work can be described as follows: using GNSS spoofing technology to control or even counter moving objects, such as unidentified aircraft, such as UAV, that may pose threats is a very effective means. However, as more and more navigation systems of unmanned aircraft are equipped with coupled GNSS/ IMU systems, which can effectively detect spoofing, it is more and more difficult for the spoofer to achieve effective spoofing. For the spoofer, the specific difficulties of spoofing are as follows: to sum up, the difficulty of spoofing the coupled system is that even if the spoofer intends to slowly change the positioning of the coupled system, they should pay close attention to whether other state parameters of the coupled system change abnormally in the process of spoofing; in addition, although the loosely coupled system itself has good anti-spoofing ability, if the coupled system is additionally equipped with other anti-spoofing techniques, the spoofer needs to consider how to introduce an appropriate amount of spoofing observation, so that the spoofing process will not raise the alarm of the coupled system.

Based on the above research background, the research motivation of this paper is to propose a spoofing algorithm that can slowly pull the positioning results of a loosely coupled GNSS/IMU system and avoid a variety of anti-spoofing techniques so as to realize successful spoofing on a loosely coupled GNSS/IMU system and further realize the effective control of the unidentified aircraft that poses threats.

The research work of this paper is briefly summarized as follows. In Section 1, the background, current situation and significance of the research are introduced. In Section 2, the influence mechanism of spoofing on loosely coupled GNSS/IMU is analyzed, including the loosely coupled GNSS/IMU system model and influence of spoofing on loosely coupled GNSS/IMU. In Section 3, a slowly varying spoofing algorithm to avoid multiple anti-spoofing techniques is proposed, after the spoofing signal completely takes over the GNSS module of the coupled system, based on the analysis of the influence mechanism of spoofing on the positioning of loosely couped GNSS/IMU, a slowly varying spoofing algorithm avoiding loosely coupled GNSS/IMU with multiple anti-spoofing techniques, a measurement deviation determination method is proposed to avoid a variety of anti-spoofing techniques, which can gradually pull the positioning results of the loosely coupled system, and successfully avoid the anti-spoofing techniques’ detection of innovation sequence monitoring and parameter rationality check so as to achieve the purpose of spoofing. In Section 4, experiments verify the effectiveness and concealment of the algorithm. In Section 5, we give a summary and outlook on the work of the paper.

## 2. Influence Mechanism of Spoofing on Loosely Coupled GNSS/IMU

### 2.1. Loosely Coupled GNSS/IMU System Model

Loosely coupled GNSS/IMU takes the error equation of GNSS and IMU as the system state equation, takes the difference of navigation information output by GNSS and IMU as the measurement, establishes the measurement equation, uses the optimal filter to fuse the two data and give the optimal estimation results, and finally feeds back to IMU for correction to realize high-precision navigation [19].

**Figure 1:**
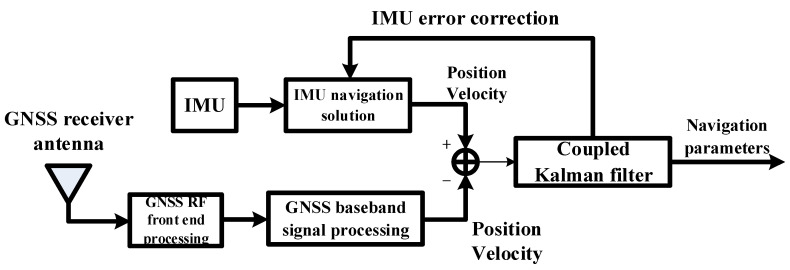
Structure block diagram of loosely coupled GNSS/IMU system.

The measurement information used by loosely coupled GNSS/IMU is position difference and velocity difference, that is, the difference between position and velocity obtained by GNSS and position and velocity calculated by IMU is used as the input of the Kalman filter, and the output is closed-loop correction. The estimation result of the Kalman filter is used to correct the IMU measurement [19]. The block diagram of the loosely coupled structure is shown in Figure 1.

Selecting the 15-dimensional IMU navigation parameter error as the state of the filter, closed-loop correction is carried out to correct the position, velocity and altitude of IMU.

### 2.2. Influence of Spoofing on Loosely Coupled GNSS/IMU

From the analysis of the composition mechanism of loosely coupled GNSS/IMU, when loosely coupled GNSS/IMU is spoofed by GNSS, firstly, the GNSS spoofing signal affects the positioning results of the GNSS output, and then affects the measured value of the Kalman filtering process of a loosely coupled system. Finally, the Kalman filter affects the error estimation of state parameters [18].

Suppose GNSS/IMU is in a normal working state before time i, and GNSS/IMU is spoofed by GNSS spoofing at time i, and then the measurement innovation vector of the system at time i is $\delta {ɀ}_{i}+\Delta {ɀ}_{i}$, and $\Delta {ɀ}_{i}$ represents the deviation of the measurement innovation vector introduced by GNSS spoofing. $\delta {ɀ}_{i}+\Delta {ɀ}_{i}$ is expressed as

(1) $$\delta {ɀ}_{i}+\Delta {ɀ}_{i}=\left[\begin{array}{c} \delta {ɀ}_{r,i}+\Delta {ɀ}_{r,i}\\ \delta {ɀ}_{v,i}+\Delta {ɀ}_{v,i} \end{array}\right]=\left[\begin{array}{c} {\hat{r}}_{GNSS}^{e}-{\hat{r}}_{IMU}^{e}+\Delta {\hat{r}}_{GNSS}^{e}\\ {\hat{v}}_{GNSS}^{e}-{\hat{v}}_{IMU}^{e}+\Delta {\hat{v}}_{GNSS}^{e} \end{array}\right]$$

In the above formula, $\Delta {\hat{r}}_{GNSS}^{e}$ represents the position deviation at time i, and $\Delta {\hat{v}}_{GNSS}^{e}$ represents the velocity deviation at time i. $\Delta {ɀ}_{r,i}$ and $\Delta {ɀ}_{v,i}$ represent the deviation of the position innovation vector and velocity innovation vector, respectively.

In the process of GNSS spoofing, $P_{i}$, $Q_{i}$, $R_{i}$ and gain matrix $K_{i}$ of Kalman filter can be approximately unchanged, and the GNSS/IMU error estimation under GNSS spoofing can be obtained as follows:

(2) $${\hat{X}}_{i}=\Phi_{i,i-1}{\hat{X}}_{i-1}+K_{i}\left(\delta {ɀ}_{i}+\Delta {ɀ}_{i}\right)$$

Since the GNSS/IMU output results are IMU navigation parameters corrected by error estimation, error estimation deviation is the system deviation of GNSS/IMU. Therefore, the system deviation expression of GNSS/IMU is

(3) $$\Delta {\hat{X}}_{i}={\hat{X}}_{i}^{\prime }-{\hat{X}}_{i}=\left\{ \begin{array}{l} K_{1}\Delta {ɀ}_{1}\\ (I-K_{i}H_{i})\Phi_{i,i-1}\Delta {\hat{X}}_{i-1}+K_{i}\Delta {ɀ}_{i} \end{array}\right.\;,\;i=2,3,\ldots$$

In the above formula, when i≥2, $\Delta {\hat{X}}_{i}$ represents the cumulative value of the GNSS/IMU system deviation.

## 3. Slowly Varying Spoofing Algorithm to Avoid Multiple Anti-Spoofing Techniques

### 3.1. Avoiding Innovation Sequence Monitoring

In the Kalman filtering process of the loosely coupled GNSS/IMU system, innovation sequence monitoring can be used to detect small anomalies in a certain period of time [19]. For the GNSS spoofing method of slowly pulling the positioning of the loosely coupled system, it is difficult for single epoch innovation detection to detect the gradually accumulated measurement error introduced by spoofing, and innovation sequence monitoring is an effective spoofing detection method to test the small and slowly accumulated deviation between the measurement and state estimation through the recent n normalized innovation vectors. Normalized innovation is defined as

(4) $$y_{i,j}=\frac{Z_{i,j}-H_{i,j}{\hat{X}}_{i/i-1}}{\sqrt{S_{i,j,j}}}$$

where $Z_{i,j}$ is the j-th component of measurement vector $Z_{i}$ at time i, $H_{i,j}$ is the j-th line component of measurement matrix at time i, ${\hat{X}}_{i/i-1}$ is the predicted value of $X_{i}$ calculated by ${\hat{X}}_{i-1}$, $Z_{i,j}-H_{i,j}{\hat{X}}_{i/i-1}$ is a measurement innovation vector of $Z_{i,j}$, $S_{i,j,j}$ represents the (j,j)-th value of the innovation covariance $S_{i}$, and $S_{i}$ is the sum of measurement noise covariance and error covariance of state estimation converted to measurement space, that is

(5) $$S_{i}=HP_{i/i-1}H^{T}+R$$

where R is the measurement noise variance matrix.

The test statistic $u_{i,j}$ is composed of the latest n measurements to detect the smaller and slowly accumulated deviation between the measurement and state estimation:

(6) $$u_{i,j}=\frac{1}{n}\sum_{i=k+1-n}^{k}y_{i,j}$$

where n is the independent samples of zero mean unit variance Gaussian distribution, and the standard deviation of its mean is $1/\sqrt{n}$. If the absolute value of $u_{i,j}$ exceeds the threshold $\frac{T_{u}}{\sqrt{n}}$, the navigation system sends an alarm and considers that the measured value is abnormal. That is, the alarm judgment criteria are

(7) $$\left\{ \begin{array}{l} \left|u_{i,j}\right|>\frac{T_{u}}{\sqrt{n}},Alarm\\ \left|u_{i,j}\right|\leq \frac{T_{u}}{\sqrt{n}},\;Not\;Alarm \end{array}\right.$$

where $T_{u}$ is the innovation threshold, which can be determined according to Gaussian distribution.

In order to make spoofing have good concealment, it should meet $\left|u_{i,j}\right|\leq \frac{T_{u}}{\sqrt{n}}$. Next, calculate the value range of single epoch measurement deviation according to the threshold of $\left|u_{i,j}\right|$. To satisfy $\left|u_{i,j}\right|\leq \frac{T_{u}}{\sqrt{n}}$, convert the problem to $\left|y_{i,j}\right|\leq \frac{T_{u}}{\sqrt{n}}$ for each epoch. In fact, satisfying $\left|y_{i,j}\right|\leq \frac{T_{u}}{\sqrt{n}}$ is a sufficient and unnecessary condition to satisfy $\left|u_{i,j}\right|\leq \frac{T_{u}}{\sqrt{n}}$. Therefore, if the single epoch satisfies $\left|y_{i,j}\right|\leq \frac{T_{u}}{\sqrt{n}}$, it must satisfy $\left|u_{i,j}\right|\leq \frac{T_{u}}{\sqrt{n}}$.

The single epoch is analyzed below. Solve $\left|y_{i,j}\right|\leq \frac{T_{u}}{\sqrt{n}}$ to obtain

(8) $$\left\{ \begin{array}{l} \left(H_{i,j}{\hat{X}}_{i/i-1}-T_{u}\sqrt{\frac{S_{i,j,j}}{n}}+{\hat{r}}_{eb,j}^{e}\right)\leq {\hat{r}}_{eaG,j}^{e}\leq \left(H_{i,j}{\hat{X}}_{i/i-1}+T_{u}\sqrt{\frac{S_{i,j,j}}{n}}+{\hat{r}}_{eb,j}^{e}\right)\\ \left(H_{i,j}{\hat{X}}_{i/i-1}-T_{u}\sqrt{\frac{S_{i,j,j}}{n}}+{\hat{v}}_{eb,j}^{e}\right)\leq {\hat{v}}_{eaG,j}^{e}\leq \left(H_{i,j}{\hat{X}}_{i/i-1}+T_{u}\sqrt{\frac{S_{i,j,j}}{n}}+{\hat{v}}_{eb,j}^{e}\right) \end{array}\right.$$

The above formula shows that when spoofing loosely coupled GNSS/IMU, in order to avoid innovation sequence monitoring, the output position ${\hat{r}}_{eaG,j}^{e}$ and velocity ${\hat{v}}_{eaG,j}^{e}$ of GNSS spoofing signal cannot change arbitrarily, and the value range of Formula (8) needs to be met.

### 3.2. Avoiding Parameter Rationality Check

Loosely coupled GNSS/IMU can detect faults and even spoofing through the parameter rationality check. The parameter rationality check includes the sensor output, navigation parameters and Kalman filter estimation [19]. Check the output of the sensors to respond to simple spoofing. Checking the navigation parameters provides additional protection. Checking the Kalman filter estimation can check slow faults and even spoofing.

Next, the spoofing algorithm to avoid the parameter rationality check is proposed from the perspective of the spoofer. Firstly, the influence of gain matrix $K_{i}$ in the Kalman filtering process on GNSS spoofing is analyzed. $K_{\infty }$ represents the stable state of $K_{i}$, and $K_{\infty }$ is the 15×6 matrix. According to Formula (3), the relationship between the system deviation of GNSS/IMU and measured deviation can be expressed in the following matrix form:

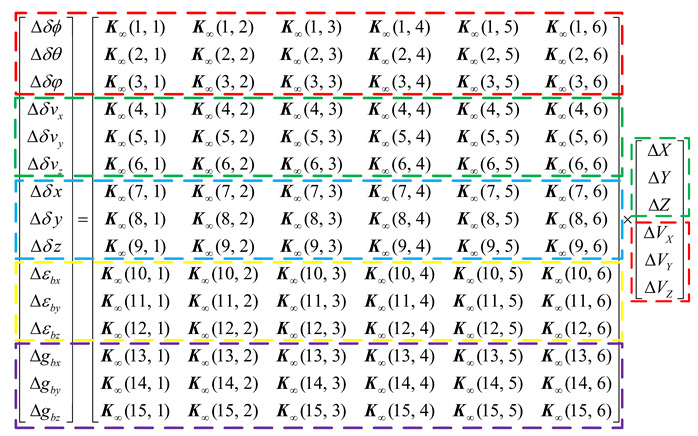
(9)
where in, ΔX, ΔY and ΔZ represent position measurement deviation under the ECEF coordinate system, and $\Delta V_{X}$, $\Delta V_{Y}$ and $\Delta V_{Z}$ represent the velocity measurement deviation under ECEF coordinate system.

For the spoofer, in order to ensure that the spoofing deviation can achieve the purpose of spoofing, GNSS spoofing shall make single epoch position deviation Δδx, Δδy and Δδz of the GNSS/IMU system introduced to target the desired position deviation $\Delta \delta x_{Ex}$, $\Delta \delta y_{Ex}$ and $\Delta \delta z_{Ex}$ as far as possible; at the same time, the constraint condition of single epoch velocity deviation $\Delta \delta v_{x}$, $\Delta \delta v_{y}$ and $\Delta \delta v_{z}$ of the GNSS/IMU system introduced into the target is the absolute value of the velocity deviation which meets the absolute value of the position deviation to which it is less than or equal. In fact, if GNSS spoofing has too much impact on the altitude estimation of the target, the expected purpose of spoofing may not be achieved. The reasons are that, on the one hand, the target (such as UAV) is more sensitive to altitude change relative to the position and velocity change, and the spoofing detection means of the target is easier to detect the abnormality of altitude estimation; on the other hand, if GNSS spoofing causes the altitude change to exceed the physical threshold of the target, it is easy to cause motion failure of the target [20] and it cannot be induced to the expected position by the spoofer. Therefore, GNSS spoofing should keep single epoch altitude angle deviation values Δδϕ, Δδθ and Δδφ of GNSS/IMU system introduced into target as normal as possible. To sum up, the formula can be expressed as

(10) $$\left\{ \begin{array}{l} \Delta \delta x\approx \Delta \delta x_{Ex}\\ \Delta \delta y\approx \Delta \delta y_{Ex}\\ \Delta \delta z\approx \Delta \delta z_{Ex} \end{array}\right.$$

If biases estimated by the loosely coupled GNSS/IMU Kalman filter exceeds five times the nominal value, it is considered that the sensor may be faulty [15]. Here, when GNSS spoofing is implemented, the bias caused by spoofing shall not exceed five times the nominal biases. That is, (9) shall meet the following constraints:

(11) $$\left\{ \begin{array}{l} \Delta \varepsilon_{bx}\leq T_{5\sigma_{1}}\\ \Delta \varepsilon_{by}\leq T_{5\sigma_{2}}\\ \Delta \varepsilon_{bz}\leq T_{5\sigma_{3}}\\ \Delta g_{bx}\leq T_{5\sigma_{4}}\\ \Delta g_{by}\leq T_{5\sigma_{5}}\\ \Delta g_{bz}\leq T_{5\sigma_{6}} \end{array}\right.$$

where $T_{5\sigma_{i}}\;,\;i=1,2,\cdots ,6$ represents five times the nominal biases of $b_{ax}$, $b_{ay}$, $b_{az}$, $b_{gx}$, and $b_{gy}$, $b_{gz}$ respectively.

According to (9), the spoofer calculates the measurement deviation required for spoofing according to the system deviation of the GNSS/IMU required for a single epoch and estimated gain matrix $K_{i}$. The position constraint in (10) is taken as the objective function of the product of gain matrix $K_{i}$ and measured value deviation; (11) is taken as the constraints to solve the optimal measured value deviation.

**Figure 2:**
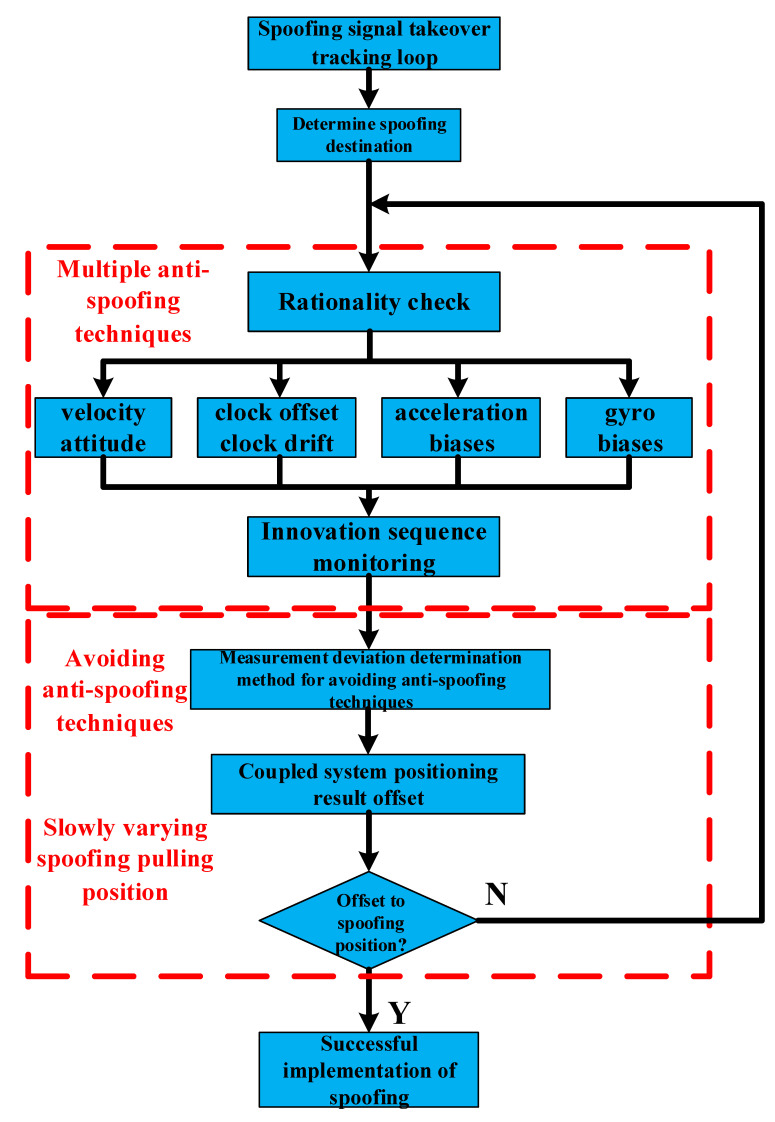
Flow chart of spoofing loosely coupled GNSS/IMU algorithm.

In combination with Section 3.1 and this section, the spoofing quantity is solved by the method in Section 3.2 to avoid the parameter rationality check. At this time, the loosely coupled GNSS/IMU system uses an innovation sequence monitoring algorithm for spoofing detection. In this case, it is necessary to reconsider spoofing quantity $\Delta {ɀ}_{i}$ to avoid innovation sequence monitoring. Based on this, $\delta z_{k,j}^{-}$ needs to satisfy (8), so (8) is also used as the constraint condition in Section 3.2 to resolve $\Delta {ɀ}_{i}$ that is not detected by the innovation sequence monitoring. In conclusion, the flow of the proposed slowly varying spoofing algorithm avoiding loosely coupled GNSS/IMU with multiple anti-spoofing techniques is shown in Figure 2.

According to Figure 2, the proposed algorithm can be described in detail as follows: after the spoofing signal completely takes over the GNSS tracking loop, the spoofer first determines the target location of spoofing. After comprehensively considering the parameter rationality check and innovation sequence monitoring of the loosely coupled system, the reasonable spoofing offset of each epoch is calculated to avoid the detection of the above anti-spoofing techniques and ensure that the positioning result of the loosely coupled GNSS/IMU is slowly changed. If the coupled system has not been spoofed to the target location after one spoofing, the spoofing algorithm will continue to be executed. Finally, it is spoofed to the target position so as to gradually achieve the purpose of spoofing.

## 4. Simulation Analysis

Based on the MATLAB simulation software provided by [19], we modified and upgraded the software to realize the experimental work of this paper. The experimental environment can well show the spoofing effect of spoofing signal on loosely coupled GNSS/IMU. In the experimental environment, the spoofing signal is fully controllable to the spoofer.

In the simulation experimental scenario, the real state of loosely coupled GNSS/IMU equipment always remains stationary at point O. The IMU in the loosely coupled system is tactical-grade IMU, and the device parameters are shown in Table 1.

In the experiment, the parameter settings of the loosely coupled GNSS/IMU Kalman filter are shown in Table 2:

At the initial time, the output positioning result of the loosely coupled system is the O point, and at this time, the loosely coupled system is taken over by the GNSS spoofing signal. The purpose of the GNSS spoofer is to offset the positioning result of the loosely coupled GNSS/IMU system to point S, which deviates from point O by 30 m to the north, 30 m to the east and 30 m down.

**Figure 3:**
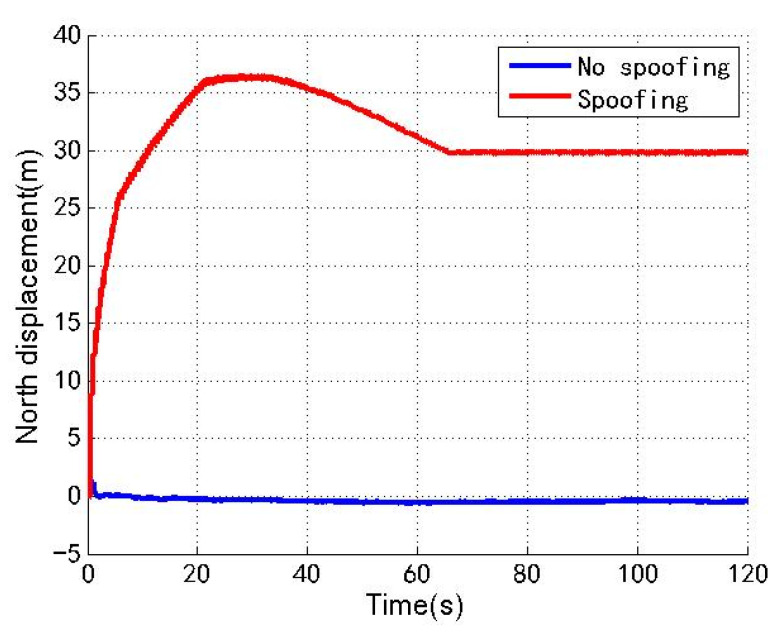
North displacement without/with spoofing.

**Figure 4:**
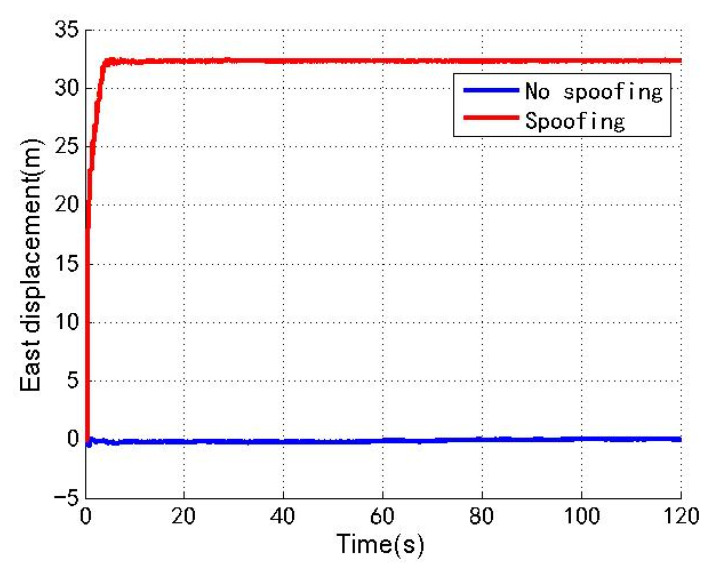
East displacement without/with spoofing.

**Figure 5:**
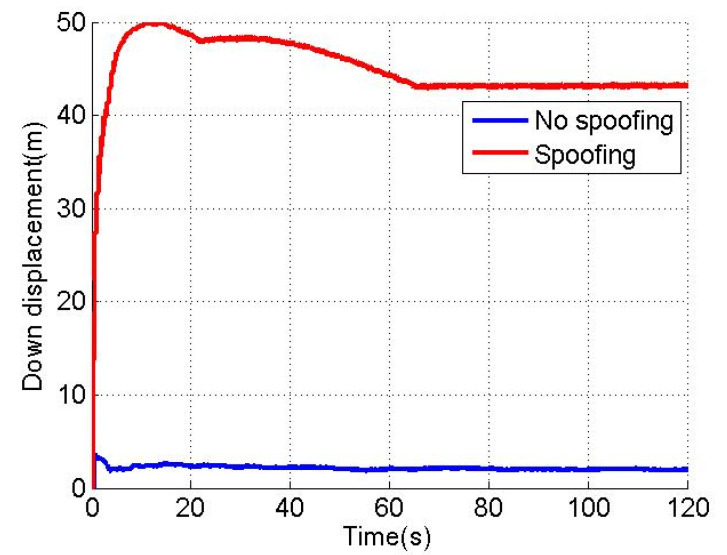
Down displacement without/with spoofing.

Figure 3, Figure 4 and Figure 5 show the north, east and down position offsets of the loosely coupled GNSS/IMU system without spoofing and with spoofing, respectively. The blue line indicates no spoofing, and the red line indicates spoofing.

As shown in Figure 3, Figure 4 and Figure 5, when the loosely coupled system is spoofed by slowly varying spoofing, the north displacement gradually shifts by 29.8 m in the period of 0–66 s, and stabilizes around 29.8 m in the period of 66–120 s; in the 0–15 s period, eastward displacement gradually shifts by 32.3 m, and in the 15–120 s period, the eastward displacement is stable around 32.3 m; in the period of 0–66 s, down displacement gradually deviates by 43.2 m; and in the period of 66–120 s, down displacement is stable around 43.2 m. In terms of the spoofing effect, the north displacement basically achieves a spoofing effect, and the error between the north displacement and expected offset is −0.2 m; the east displacement basically achieves a spoofing effect, and the error between the east displacement and expected displacement is 2.3 m; the down ground displacement also basically achieves a spoofing effect, but the effect is slightly worse than that in the north and east directions, and the error with the expected offset is 13.2 m.

**Figure 6:**
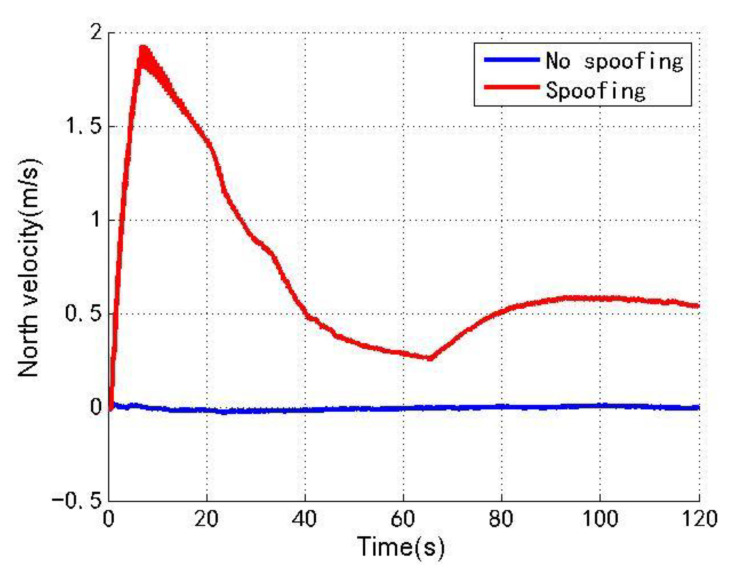
North velocity without/with spoofing.

**Figure 7:**
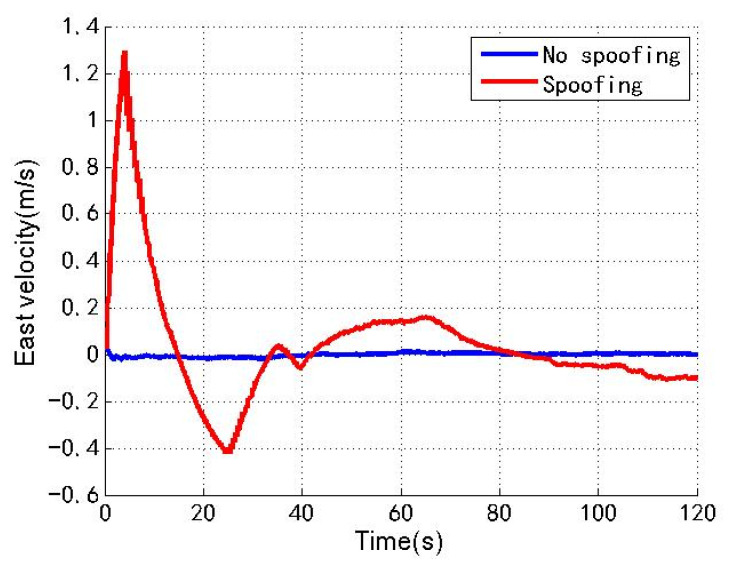
East velocity without/with spoofing.

**Figure 8:**
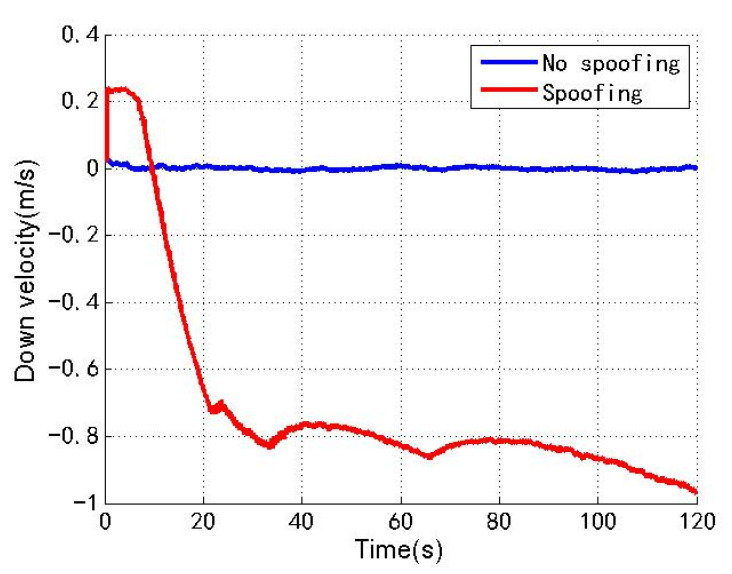
Down velocity without/with spoofing.

Figure 6, Figure 7 and Figure 8 show the north, east and down velocities of the loosely coupled GNSS/IMU system without spoofing and with spoofing, respectively. The blue line indicates no spoofing, and the red line indicates spoofing.

As shown in Figure 6, Figure 7 and Figure 8, when the loosely coupled system is spoofed by slowly varying spoofing, although there is a small fluctuation in the north velocity compared with the case without spoofing, it can always remain greater than −0.02 m/s and less than 1.94 m/s; although the east velocity fluctuates slightly, it can always remain greater than −0.43 m/s and less than 1.3 m/s; although the down velocity fluctuates slightly, it can always remain greater than −0.98 m/s and less than 0.24 m/s. To sum up, the velocity change of the loosely coupled system conforms to the parameter rationality check, and is also close to the velocity change without spoofing.

**Figure 9:**
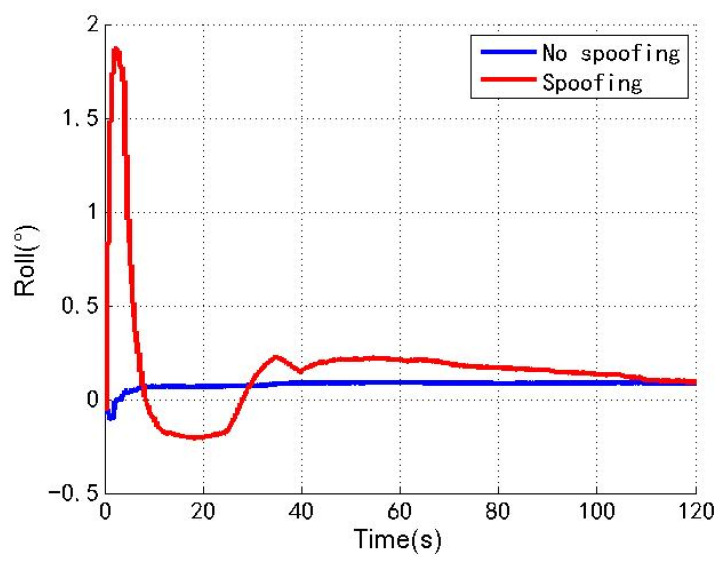
Roll angle without/with spoofing.

**Figure 10:**
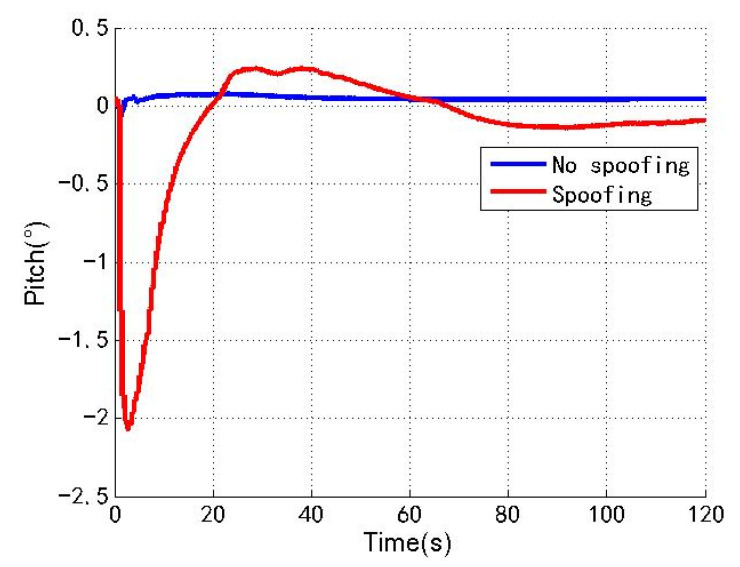
Pitch angle without/with spoofing.

**Figure 11:**
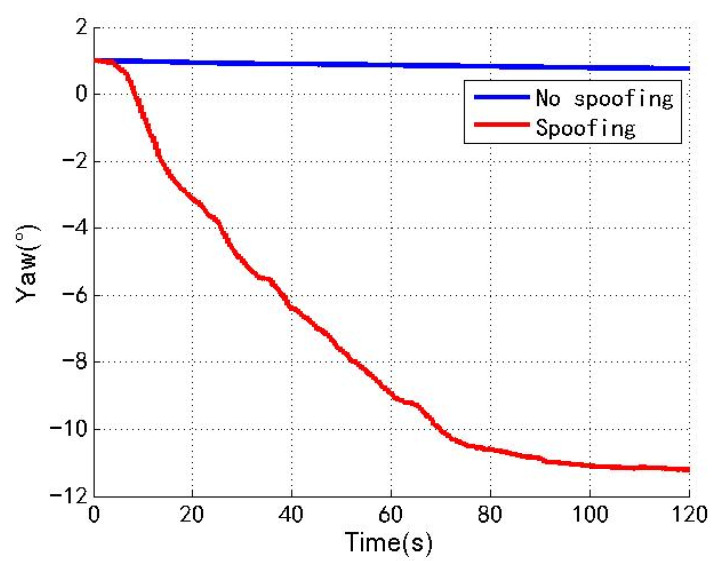
Yaw angle without/with spoofing.

Figure 9, Figure 10 and Figure 11 show the changes of the roll angle, pitch angle and yaw angle of the loosely coupled GNSS/IMU system without spoofing and with spoofing, respectively. The blue line indicates no spoofing, and the red line indicates spoofing.

As shown in Figure 9, Figure 10 and Figure 11, when the loosely coupled system is spoofed by slowly varying spoofing, although the roll angle fluctuates slightly compared with the case without spoofing, it can always remain greater than −0.21° and less than 1.88°; although the pitch angle fluctuates slightly, it can always remain greater than −2.07° and less than 0.25°; although the yaw angle fluctuates slightly, it can always remain greater than −11.21° and less than 1.01°. To sum up, the change of the loosely coupled altitude angle is also close to the change of the altitude angle without spoofing.

**Figure 12:**
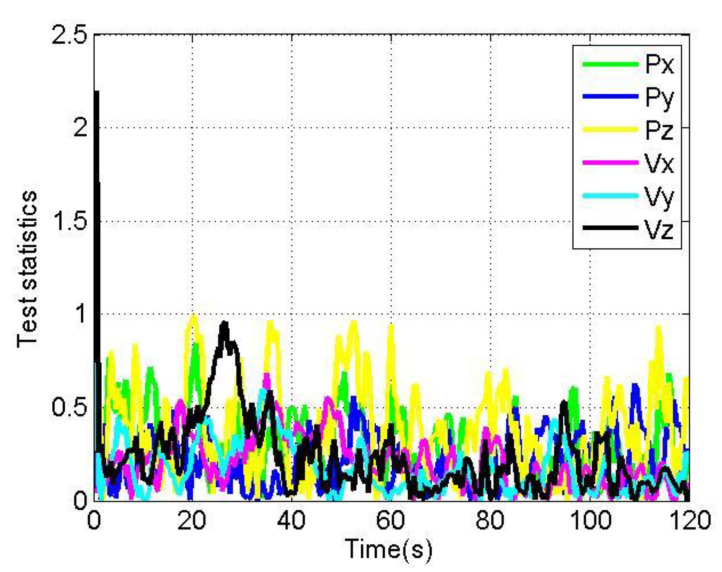
Test statistics without spoofing.

**Figure 13:**
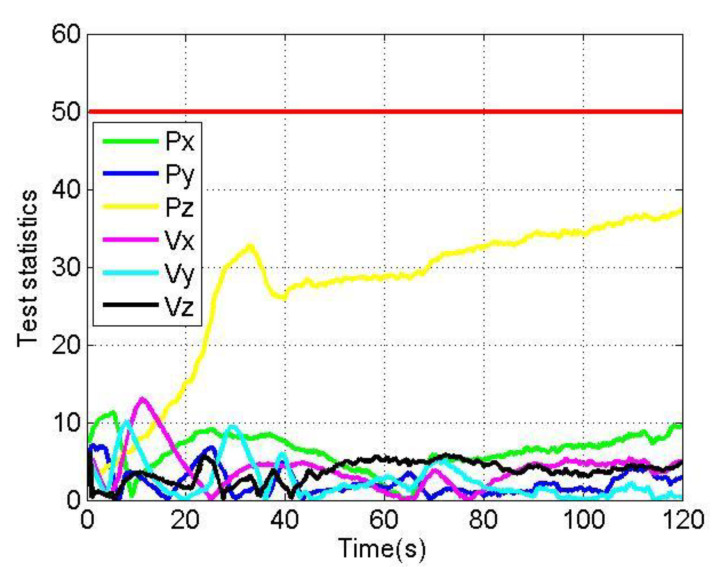
Test statistics with spoofing.

Figure 12 and Figure 13 show the changes of each test statistic $\left|\sqrt{n}*y_{i,j}\right|$ of the loosely coupled GNSS/IMU system without spoofing and with spoofing, respectively. Here, the threshold $T_{u}$ is set to 50. Px, Py, Pz, Vx, Vy and Vz respectively represent the test statistics of the position in the X direction, Y direction and Z direction; the test statistics of the velocity in the X direction, Y direction and Z direction in the ECEF coordinate system; and the red line represents the alarm threshold line.

The test statistics in Figure 12 and Figure 13 are analyzed, and the results are shown in Table 3.

As shown in Figure 12 and Figure 13, when loosely coupled GNSS/IMU is spoofed by slowly varying spoofing, although the test statistics of the loosely coupled system increase compared with the case without spoofing, they do not exceed the alarm threshold. To sum up, when the loosely coupled system is spoofed by slowly spoofing, its test statistics will not raise an alarm.

**Figure 14:**
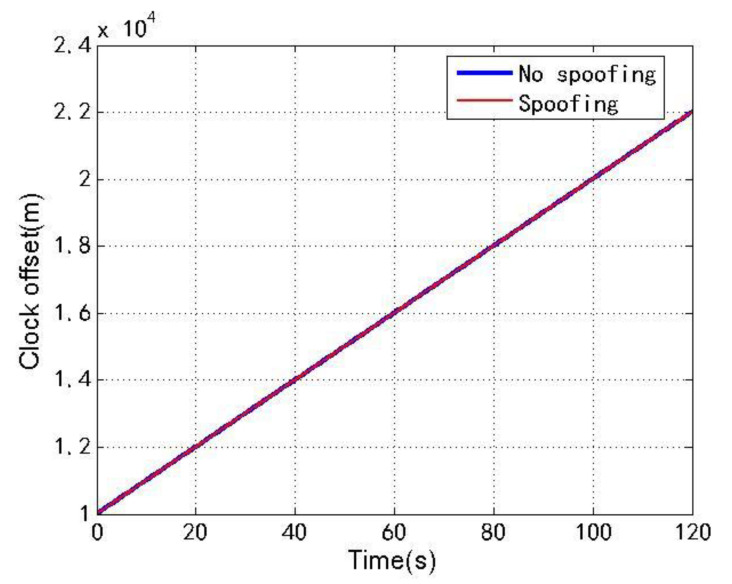
Clock offset without/with spoofing.

**Figure 15:**
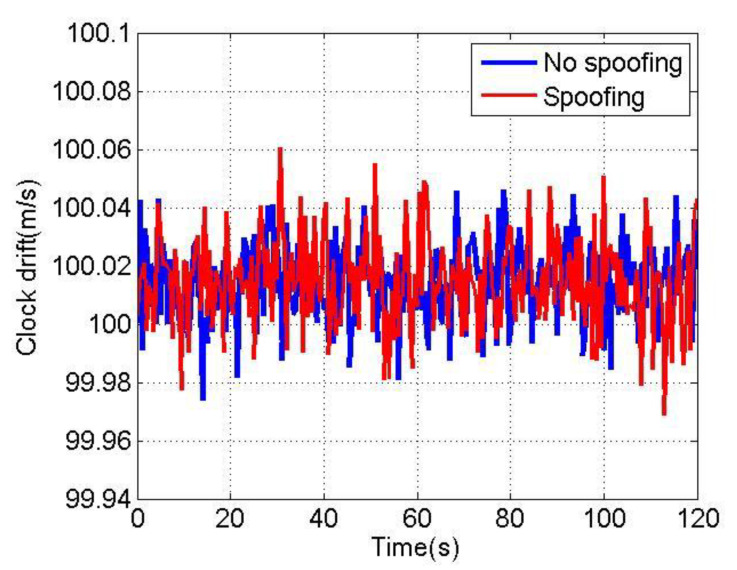
Clock drift without/with spoofing.

Figure 14 and Figure 15 show the changes of the clock offset estimation and clock drift estimation of loosely coupled GNSS/IMU without spoofing and with spoofing, respectively. The blue line indicates no spoofing, and the red line indicates spoofing.

As shown in Figure 14 and Figure 15, when the loosely coupled system is spoofed by slowly varying spoofing, the estimated loosely coupled clock offset is close to the same, compared with the case without spoofing; although the estimated clock drift fluctuates slightly, it can always remain greater than 99.7 m/s and less than 100.1 m/s. To sum up, the changes of the loosely coupled clock offset estimation and clock drift estimation are also close to those without spoofing.

**Figure 16:**
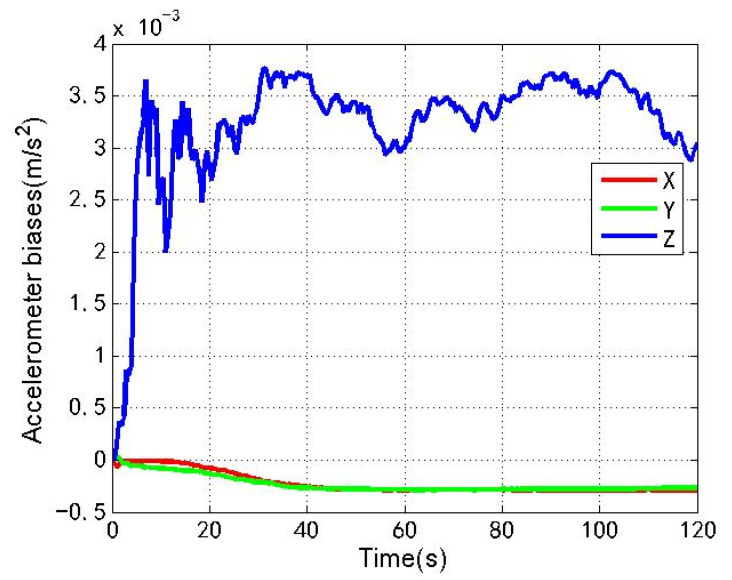
Accelerometer biases without spoofing.

**Figure 17:**
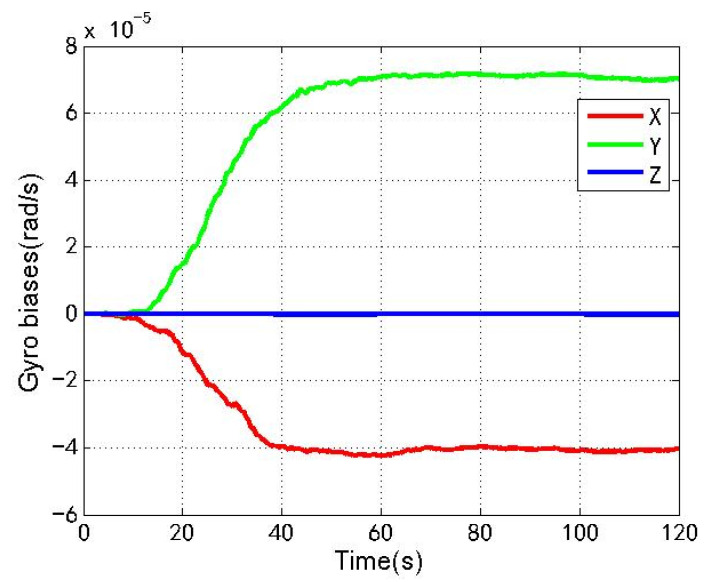
Gyro biases without spoofing.

Figure 16 and Figure 17 show the changes of the accelerometer biases estimation and gyro biases estimation of the loosely coupled GNSS/IMU system without spoofing. The red line, green line and blue line respectively represent the X, Y and Z axis directions along the body coordinate.

**Figure 18:**
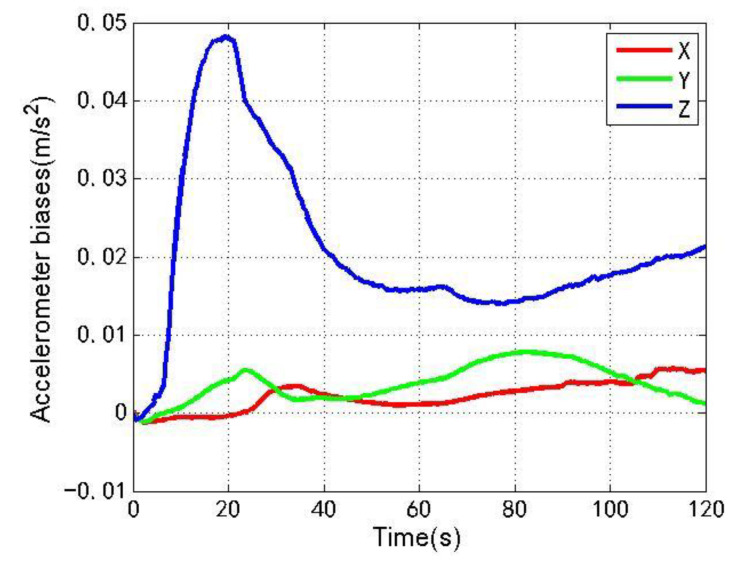
Accelerometer biases with spoofing.

**Figure 19:**
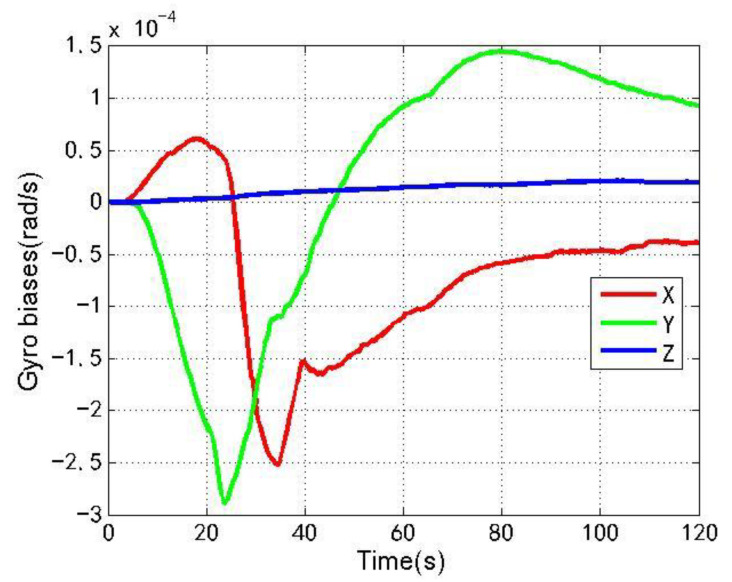
Gyro biases with spoofing.

Figure 18 and Figure 19 show the changes of the accelerometer biases estimation and gyro biases estimation of the loosely coupled GNSS/IMU system with spoofing. The red line, green line and blue line respectively represent the X, Y and Z axis directions along the body coordinate.

As shown in Figure 16, Figure 17, Figure 18 and Figure 19, when the loosely coupled system is spoofed by slowly varying spoofing, compared with the case without spoofing, although the estimated acceleration biases fluctuate slightly in the X direction, it can always remain greater than $-1.2\;\times {\;10}^{-3}{\;m/s}^{2}$ and less than $5.8\;\times {\;10}^{-3}{\;m/s}^{2}$; although there is a small fluctuation in the Y direction, it can always remain greater than $-1.1\;\times {\;10}^{-3}{\;m/s}^{2}$ and less than $7.9\;\times {\;10}^{-3}{\;m/s}^{2}$; and although there is a small fluctuation in the Z direction, it can always remain greater than $-1.0\;\times {\;10}^{-3}{\;m/s}^{2}$ and less than $4.8\;\times {\;10}^{-2}{\;m/s}^{2}$. Although the estimated gyro biases fluctuate slightly in the X direction, it can always remain greater than $-2.6\;\times {\;10}^{-4}\;\mathrm{rad}/s$ and less than $6.1\;\times {\;10}^{-5}\;\mathrm{rad}/s$; although there is a small fluctuation in the Y direction, it can always remain greater than $-2.9\;\times {\;10}^{-4}\;\mathrm{rad}/s$ and less than $1.5\;\times {\;10}^{-4}\;\mathrm{rad}/s$; and although there is a small fluctuation in the Z direction, it can always remain greater than 0 and less than $2.1\;\times {\;10}^{-5}\;\mathrm{rad}/s$. To sum up, the variation of the biases estimation of the accelerometer and gyro is also close to the variation without spoofing.

Based on the above experimental analysis, in terms of the spoofing effect, the north displacement completely achieves the spoofing effect, and errors with the expected offset are −0.2 m; the east displacement basically achieves the spoofing effect, and the error with expected offset is 2.3 m; the down displacement also basically achieves the spoofing effect, but the effect is slightly worse than that in the north and east directions, and error with the expected offset is 13.2 m. When slowly varying spoofing is applied to a loosely coupled system, the velocity change of the loosely coupled system is close to the velocity change of that without spoofing, and the change of the altitude angle is also close to the change of the altitude angle without spoofing. At the same time, its test statistics will not raise an alarm, and the changes of the accelerometer bias estimation and gyro bias estimation of a tightly coupled system are also close to the change without spoofing.

## 5. Conclusions and Future Work

In order to effectively counter the non-cooperative target of an assembly integrated navigation system by using spoofing technology, a new spoofing algorithm needs to be proposed. In order to effectively counter the non-cooperative target of assembling a loosely coupled GNSS/IMU by using GNSS spoofing, based on the analysis of the influence mechanism of spoofing on the positioning of loosely coupled GNSS/IMU, a slowly varying spoofing algorithm to avoid loosely coupled GNSS/IMU with multiple anti-spoofing techniques is proposed, and a measurement deviation determination method to avoid multiple anti-spoofing techniques is proposed, which can gradually pull the positioning results of coupled system and successfully avoid anti-spoofing techniques’ detection of innovation sequence monitoring and a parameter rationality check. The experimental results show that the proposed algorithm gradually changes the positioning of the loosely coupled GNSS/IMU; the north and east displacements achieve the purpose of spoofing; and the errors with the expected offset are −0.2 m and 2.3 m, respectively. The down displacement also basically achieves the purpose of spoofing, and the error with the expected offset is 13.2 m. At the same time, it avoids the detection of multiple anti-spoofing techniques, does not trigger the system alarm, and realizes the purpose of spoofing, thus the effectiveness and high concealment of the spoofing algorithm are verified. The research results provide an effective solution for the target equipped with a loosely coupled GNSS/IMU system to implement GNSS spoofing. On the other hand, it also provides reference for a loosely coupled GNSS/IMU system to detect and suppress GNSS spoofing.

The research work of this paper, which needs to be improved in the follow-up research, may be that the spoofing in the experiment is positioning spoofing, and the set spoofing destination is not very complex. Future research work will focus on, first, how to spoof the more complex coupled system of GNSS and IMU, and second, how to spoof more complex anti-spoofing techniques to deal with the threat of unknown moving objects.

## Figures and Tables

**Table 1:** Parameters of tactical-grade IMU.

Parameter	Value	Unit
IMU accelerometer biases	[900; −1300; 800]	µg
IMU accelerometer noise root PSD	100	µg/$\sqrt{\mathrm{Hz}}$
IMU gyro biases	[−9; −13; −8]	°/h
IMU gyro noise root PSD	0.01	°/$\sqrt{h}$

**Table 2:** Parameters setting of Kalman filter for loosely coupled system.

Parameter	Value	Unit
Accelerometer bias random walk PSD	10^−7^	m^2^/s^5^
Gyro bias random walk PSD	2 × 10^−12^	rad^2^/s^3^
Position measurement noise SD	2.5	m
Velocity measurement noise SD	0.1	m/s

**Table 3:** Analysis of test statistics of loosely coupled without/with spoofing.

Test Statistics	Without/With Spoofing	Average	Maximum
Px	Without With	0.29 6.23	0.85 11.27
Py	Without With	0.22 2.18	0.63 7.05
Pz	Without With	0.37 27.12	0.99 37.39
Vx	Without With	0.20 3.86	0.68 13.08
Vy	Without With	0.18 2.44	0.80 10.21
Vz	Without With	0.23 3.60	2.20 6.32

## Data Availability

The data used in this research are not publicly available.
